# 762 A Systematic Review of Surgical Simulation in Burn Surgery: Education, Assessment, and Management

**DOI:** 10.1093/jbcr/irad045.237

**Published:** 2023-05-15

**Authors:** Idean Roohani, Tayla Moshal, Devon O'Brien, Christian Jimenez, Zachary Collier, Katelyn Kondra, Haig Yenikomshian, Justin Gillenwater, Joseph Carey

**Affiliations:** Division of Plastic and Reconstructive Surgery, Keck School of Medicine of USC, Los Angeles, CA, Los Angeles, California; Division of Plastic and Reconstructive Surgery, Keck School of Medicine of USC, Los Angeles, California; Division of Plastic and Reconstructive Surgery, Keck School of Medicine of USC, South Pasadena, California; Division of Plastic and Reconstructive Surgery, Keck School of Medicine of USC, Los Angeles, CA, Los Angeles, California; Division of Plastic and Reconstructive Surgery, Keck School of Medicine of USC, Los Angeles, CA, Los Angeles, California; Keck Medicine of USC, Los Angeles, California; University of Southern California, Los Angeles, California; Keck Medicine of USC, Los Angeles, California; Division of Plastic and Reconstructive Surgery, Keck School of Medicine of USC, Los Angeles, CA, Los Angeles, California; Division of Plastic and Reconstructive Surgery, Keck School of Medicine of USC, Los Angeles, CA, Los Angeles, California; Division of Plastic and Reconstructive Surgery, Keck School of Medicine of USC, Los Angeles, California; Division of Plastic and Reconstructive Surgery, Keck School of Medicine of USC, South Pasadena, California; Division of Plastic and Reconstructive Surgery, Keck School of Medicine of USC, Los Angeles, CA, Los Angeles, California; Division of Plastic and Reconstructive Surgery, Keck School of Medicine of USC, Los Angeles, CA, Los Angeles, California; Keck Medicine of USC, Los Angeles, California; University of Southern California, Los Angeles, California; Keck Medicine of USC, Los Angeles, California; Division of Plastic and Reconstructive Surgery, Keck School of Medicine of USC, Los Angeles, CA, Los Angeles, California; Division of Plastic and Reconstructive Surgery, Keck School of Medicine of USC, Los Angeles, CA, Los Angeles, California; Division of Plastic and Reconstructive Surgery, Keck School of Medicine of USC, Los Angeles, California; Division of Plastic and Reconstructive Surgery, Keck School of Medicine of USC, South Pasadena, California; Division of Plastic and Reconstructive Surgery, Keck School of Medicine of USC, Los Angeles, CA, Los Angeles, California; Division of Plastic and Reconstructive Surgery, Keck School of Medicine of USC, Los Angeles, CA, Los Angeles, California; Keck Medicine of USC, Los Angeles, California; University of Southern California, Los Angeles, California; Keck Medicine of USC, Los Angeles, California; Division of Plastic and Reconstructive Surgery, Keck School of Medicine of USC, Los Angeles, CA, Los Angeles, California; Division of Plastic and Reconstructive Surgery, Keck School of Medicine of USC, Los Angeles, CA, Los Angeles, California; Division of Plastic and Reconstructive Surgery, Keck School of Medicine of USC, Los Angeles, California; Division of Plastic and Reconstructive Surgery, Keck School of Medicine of USC, South Pasadena, California; Division of Plastic and Reconstructive Surgery, Keck School of Medicine of USC, Los Angeles, CA, Los Angeles, California; Division of Plastic and Reconstructive Surgery, Keck School of Medicine of USC, Los Angeles, CA, Los Angeles, California; Keck Medicine of USC, Los Angeles, California; University of Southern California, Los Angeles, California; Keck Medicine of USC, Los Angeles, California; Division of Plastic and Reconstructive Surgery, Keck School of Medicine of USC, Los Angeles, CA, Los Angeles, California; Division of Plastic and Reconstructive Surgery, Keck School of Medicine of USC, Los Angeles, CA, Los Angeles, California; Division of Plastic and Reconstructive Surgery, Keck School of Medicine of USC, Los Angeles, California; Division of Plastic and Reconstructive Surgery, Keck School of Medicine of USC, South Pasadena, California; Division of Plastic and Reconstructive Surgery, Keck School of Medicine of USC, Los Angeles, CA, Los Angeles, California; Division of Plastic and Reconstructive Surgery, Keck School of Medicine of USC, Los Angeles, CA, Los Angeles, California; Keck Medicine of USC, Los Angeles, California; University of Southern California, Los Angeles, California; Keck Medicine of USC, Los Angeles, California; Division of Plastic and Reconstructive Surgery, Keck School of Medicine of USC, Los Angeles, CA, Los Angeles, California; Division of Plastic and Reconstructive Surgery, Keck School of Medicine of USC, Los Angeles, CA, Los Angeles, California; Division of Plastic and Reconstructive Surgery, Keck School of Medicine of USC, Los Angeles, California; Division of Plastic and Reconstructive Surgery, Keck School of Medicine of USC, South Pasadena, California; Division of Plastic and Reconstructive Surgery, Keck School of Medicine of USC, Los Angeles, CA, Los Angeles, California; Division of Plastic and Reconstructive Surgery, Keck School of Medicine of USC, Los Angeles, CA, Los Angeles, California; Keck Medicine of USC, Los Angeles, California; University of Southern California, Los Angeles, California; Keck Medicine of USC, Los Angeles, California; Division of Plastic and Reconstructive Surgery, Keck School of Medicine of USC, Los Angeles, CA, Los Angeles, California; Division of Plastic and Reconstructive Surgery, Keck School of Medicine of USC, Los Angeles, CA, Los Angeles, California; Division of Plastic and Reconstructive Surgery, Keck School of Medicine of USC, Los Angeles, California; Division of Plastic and Reconstructive Surgery, Keck School of Medicine of USC, South Pasadena, California; Division of Plastic and Reconstructive Surgery, Keck School of Medicine of USC, Los Angeles, CA, Los Angeles, California; Division of Plastic and Reconstructive Surgery, Keck School of Medicine of USC, Los Angeles, CA, Los Angeles, California; Keck Medicine of USC, Los Angeles, California; University of Southern California, Los Angeles, California; Keck Medicine of USC, Los Angeles, California; Division of Plastic and Reconstructive Surgery, Keck School of Medicine of USC, Los Angeles, CA, Los Angeles, California; Division of Plastic and Reconstructive Surgery, Keck School of Medicine of USC, Los Angeles, CA, Los Angeles, California; Division of Plastic and Reconstructive Surgery, Keck School of Medicine of USC, Los Angeles, California; Division of Plastic and Reconstructive Surgery, Keck School of Medicine of USC, South Pasadena, California; Division of Plastic and Reconstructive Surgery, Keck School of Medicine of USC, Los Angeles, CA, Los Angeles, California; Division of Plastic and Reconstructive Surgery, Keck School of Medicine of USC, Los Angeles, CA, Los Angeles, California; Keck Medicine of USC, Los Angeles, California; University of Southern California, Los Angeles, California; Keck Medicine of USC, Los Angeles, California; Division of Plastic and Reconstructive Surgery, Keck School of Medicine of USC, Los Angeles, CA, Los Angeles, California

## Abstract

**Introduction:**

Proper assessment and management of burn patients requires practical knowledge and timely interventions in a high-stakes setting. Although much of burn care emphasizes critical care, nuances exist among this patient population, creating challenges for surgeons-in-training. As a result, low- and high-fidelity burn simulations have become increasingly popular means to educate burn care physicians and their teams in a safe environment without compromising care or endangering patients. The aim of this report is to systematically review the literature for existing simulations related to burn care.

**Methods:**

A systematic review was conducted using the following databases: PubMed, Scopus, Embase, Web of Science, and Cochrane. Inclusion criteria were peer-reviewed articles about surgical simulation models related to burn care and surgery. Skills and techniques taught, assessment methods, model type, fidelity, and equipment were extracted from included studies. Conference proceedings, reviews, non-English and non-burn-specific literature were excluded.

**Results:**

Our search criteria identified 2,585 articles, 16 of which met inclusion criteria. Simulation methods included 7 simulation courses (43.8%), 6 synthetic benchtop models (37.5%), 2 augmented/virtual reality interfaces (12.5%), and 2 iOS applications (12.5%). There were no burn-specific animal or cadaveric models. Model fidelities included 9 (56.3%) high-, 3 (18.8%) medium-, and 4 (25.0%) low-fidelity. The most common topics included in the simulations were burn wound assessment/management (n=10, 62.5%), escharotomy (n=6, 37.5%), fluid management (n=2, 12.5%), electrical burns (n=1, 6.2%), skin graft harvest (n=1, 6.2%), tangential excision (n=1, 6.2%), and cricothyrotomy (n=1, 6.2%).

**Conclusions:**

Burn management requires an expansive breadth of intensive care knowledge and surgical expertise combined with a distinct, burn-specific skill set. A paucity exists in the literature for augmented/virtual reality and animal/cadaver models unique to burn care and surgery. Our review identified a need to expand simulator options to improve training in skin grafting, burn reconstruction, and burns-specific intensive care management.

**Applicability of Research to Practice:**

Given the rapidly expanding emphasis on remote learning in the post-COVID era, developing accessible surgical simulation infrastructure adjacent to clinical training may better prepare global burn providers for the complexity of burn care.

